# Circadian Chimeric Mice Reveal an Interplay Between the Suprachiasmatic Nucleus and Local Brain Clocks in the Control of Sleep and Memory

**DOI:** 10.3389/fnins.2021.639281

**Published:** 2021-02-19

**Authors:** Elizabeth Susan Maywood, Johanna Elizabeth Chesham, Raphaelle Winsky-Sommerer, Nicola Jane Smyllie, Michael Harvey Hastings

**Affiliations:** ^1^Division of Neurobiology, MRC Laboratory of Molecular Biology, Cambridge, United Kingdom; ^2^Surrey Sleep Research Centre, Faculty of Health and Medical Sciences, University of Surrey, Guildford, United Kingdom

**Keywords:** REM sleep, NREM sleep, electroencephalogram, circadian misalignment, suprachiasmatic nucleus

## Abstract

Sleep is regulated by circadian and homeostatic processes. Whereas the suprachiasmatic nucleus (SCN) is viewed as the principal mediator of circadian control, the contributions of sub-ordinate local circadian clocks distributed across the brain are unknown. To test whether the SCN and local brain clocks interact to regulate sleep, we used intersectional genetics to create temporally chimeric CK1ε *Tau* mice, in which dopamine 1a receptor (*Drd1a*)-expressing cells, a powerful pacemaking sub-population of the SCN, had a cell-autonomous circadian period of 24 h whereas the rest of the SCN and the brain had intrinsic periods of 20 h. We compared these mice with non-chimeric 24 h wild-types (WT) and 20 h CK1ε *Tau* mutants. The periods of the SCN *ex vivo* and the *in vivo* circadian behavior of chimeric mice were 24 h, as with WT, whereas other tissues in the chimeras had *ex vivo* periods of 20 h, as did all tissues from *Tau* mice. Nevertheless, the chimeric SCN imposed its 24 h period on the circadian patterning of sleep. When compared to 24 h WT and 20 h *Tau* mice, however, the sleep/wake cycle of chimeric mice under free-running conditions was disrupted, with more fragmented sleep and an increased number of short NREMS and REMS episodes. Even though the chimeras could entrain to 20 h light:dark cycles, the onset of activity and wakefulness was delayed, suggesting that SCN *Drd1a*-Cre cells regulate the sleep/wake transition. Chimeric mice also displayed a blunted homeostatic response to 6 h sleep deprivation (SD) with an impaired ability to recover lost sleep. Furthermore, sleep-dependent memory was compromised in chimeras, which performed significantly worse than 24 h WT and 20 h *Tau* mice. These results demonstrate a central role for the circadian clocks of SCN *Drd1a* cells in circadian sleep regulation, but they also indicate a role for extra-SCN clocks. In circumstances where the SCN and sub-ordinate local clocks are temporally mis-aligned, the SCN can maintain overall circadian control, but sleep consolidation and recovery from SD are compromised. The importance of temporal alignment between SCN and extra-SCN clocks for maintaining vigilance state, restorative sleep and memory may have relevance to circadian misalignment in humans, with environmental (e.g., shift work) causes.

## Introduction

Sleep is a vital function of the brain, implicated in various central and peripheral processes, including synaptic plasticity, brain metabolite clearance and immune competence (Vassalli and Dijk, 2009; Xie et al., 2013; de Vivo et al., 2017; Diering et al., 2017). Consequently, disruption of sleep is associated with a broad range of impairments such as compromised alertness, attention and memory (Lo et al., 2012; Chellappa et al., 2019) as well as chronic conditions such as psychiatric disorders. The most studied mathematical model of sleep regulation posits the interaction of two processes; a homeostatic process that tracks sleep need as a function of sleep–wake history, and a circadian process that ensures the appropriate timing of sleep relative to the anticipated light-dark cycle (Borbely and Achermann, 1999; Skeldon et al., 2014; Borbely et al., 2016).

The identity of the sleep homeostat remains largely unknown. In contrast, the principal circadian clock of the brain, the suprachiasmatic nucleus (SCN), has been conventionally viewed as the mediator of circadian control to sleep (Hastings et al., 2008). At the molecular level, the SCN clock consists of self-sustaining oscillatory transcriptional/post-translational feedback loops (TTFL) in which *Period* (*Per*) and *Cryptochrome* (*Cry*) genes are trans-activated by CLOCK and BMAL1 heterodimers acting at E-box regulatory sequences. Following their accumulation over several hours, the encoded PER and CRY proteins inhibit E-box activation, closing the feedback loop (Hastings et al., 2014). Progressive degradation of these negative factors ultimately allows the cycle to start again, ca. 24 h after its previous initiation. Importantly, this circadian mechanism is present in practically all tissues, including local brain areas (Hastings et al., 2008). The role of the SCN is to maintain and synchronize these local clocks to ensure coherent daily rhythms of behavior and metabolism (Hastings et al., 2007).

Initiation and maintenance of vigilance states is mediated by distributed neural networks, including centers in the brainstem and ventral forebrain, which themselves project to higher sub-cortical and cortical areas (Luppi and Fort, 2019). In turn, activity within these effector pathways is regulated indirectly and directly by the SCN such that the daily 24 h sleep–wake cycle is timed appropriately for the particular species. The discovery of local circadian clocks across the brain, including regions involved in the regulation of the 24 h sleep–wake cycle and cognition (Kyriacou and Hastings, 2010) therefore provides a new perspective on the two-process model of sleep regulation. Is the SCN the only locus of circadian control to sleep, or do these local brain clocks also contribute?

Local deletion of BMAL1 demonstrated a potential role in sleep–wake regulation for the clock of histaminergic neurons of the mouse tuberomammillary nucleus (Yu et al., 2014). Deletion of BMAL1 removes circadian function in cells, but it may also compromise non-circadian transcriptional targets, not least neurotransmitters and neuropeptides (Yu et al., 2014, 2019; Lee et al., 2015; Mieda et al., 2015). As an alternative approach to testing the role of extra-SCN clocks, we created temporally chimeric mice in which circadian timekeeping was intact in all cells but with contrasting genetically specified periods in the SCN and local clocks. If the SCN alone mediates circadian control, i.e., local clocks make no contribution (the null hypothesis), sleep timing, structure and regulation in temporally chimeric mice would be the same as in control mice. In contrast, if local clocks do contribute to the circadian control of sleep, sleep parameters would be altered in the chimeras to reflect the temporal incoherence. We therefore used intersectional genetics to delete a floxed exon of casein kinase 1 epsilon (CK1ε) in Drd1a-Cre cells, and created circadian chimeric mice whereby Cre-expressing cells had a period of 24 h whereas the rest of the SCN and the brain had a period of 20 h (Smyllie et al., 2016). This enabled us to examine the interaction between the SCN and local clocks on the circadian and homeostatic control of sleep regulation.

## Materials and Methods

### Animals and Housing

All experiments were conducted in accordance with the UK Animals (Scientific Procedures) Act of 1986, with local ethical approval (MRC LMB, AWERB). *Drd1a-Cre* mice (Tg(Drd1-cre)EY266Gsat/Mmucd, RRID:MMRRC_030779-UCD) were purchased from the GENSAT project (Rockefeller University, New York, United States), through the Mutant Mouse Regional Resource Centers (MMRRC, United States). *ROSA-YFP* mice were provided by Dr. A. McKenzie (MRC LMB). Temporally chimeric mice were created by crossing *Drd1a-Cre, ROSA26-EYFP* mice with homozygotes for the floxed *CK1*ε *Tau* allele (Smyllie et al., 2016). All mice expressed the PER2::LUC bioluminescent reporter (Yoo et al., 2005) and had a C57/BL/6J background. This generated four genotypes: CRE-negative, *CK1*ε*^*WT/WT*^*; CRE-positive, *CK1*ε*^*WT/WT*^*; CRE-negative, *CK1*ε*^*Tau/Tau*^* (*Tau* controls); CRE-positive, *CK1*ε*^*Tau/Tau*^* (chimera). The first two groups were combined as WT, in light of no differences between them. Males aged 4–6 months old were used to avoid the estrous modulation of activity patterns.

**FIGURE 1:**
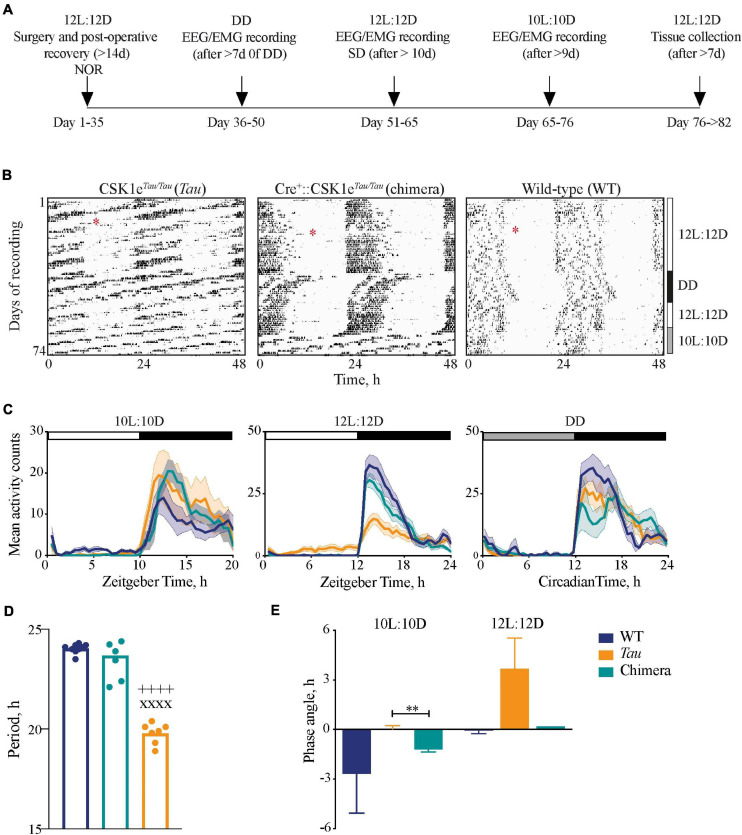
Impact of circadian chimerism on locomotor activity. **(A)** Schematic showing the timeline of the experimental protocol (NOR, novel object recognition task; SD, sleep deprivation). **(B)** Representative double-plotted wheel-running actograms from *Tau* control (left), chimeric (middle) and WT (right) mice in LD (12L:12D and 10L:10D) and constant (DD) conditions, as indicated to the right. Surgery for implantation of telemetry device is denoted by the red asterisks. **(C)** Mean (±SEM) activity profiles of WT (blue), *Tau* (orange), and chimeric (green) mice in 10L:10D (left), 12L:12D (middle), and DD (right) (*n* = 6–9). **(D)** Mean (±SEM) and individual periods of locomotor activity from WT (blue, *n* = 9), *Tau* (orange, *n* = 7), and chimeric (green, *n* = 7) mice in DD (1xANOVA *post hoc* Tukey’s multiple comparisons test xxxx*p* < 0.0001 *Tau* vs. WT, ++++*p* < 0.0001 *Tau* vs. chimera). **(E)** Mean (±SEM) phase angle of entrainment to 10L:10D and 12L:12D photoschedules (*n* = 6–7; unpaired *t*-test **p < 0.005 *Tau* vs. chimera).

Mice were housed individually and their activity patterns were assessed using running-wheels and passive infrared movement detectors. Food and water were provided *ad libitum*. Mice were entrained to a 12 h light:12 h dim red light cycle (LD) for at least 10 days before and after surgery (see below) then transferred to a schedule of continuous dim red light (5.5 ± 0.4lux; DD) for 14 days for assessment of sleep under free-running conditions (started after > 7 days of DD). All mice were confirmed to have a stable “revertant” phenotype (Smyllie et al., 2016). Mice were then re-entrained to a 12L:12D cycle for assessment of their response to sleep deprivation (SD). They were then placed into a 10L:10D photoschedule for further assessment of sleep-wake cycles (Smyllie et al., 2016) (Figure 1A). In all our studies, Zeitgeber time (ZT) 0 denotes the time of lights on and ZT12 lights off in a light/dark cycle, whereas circadian time (CT) 0 denotes the start of subjective day and CT12 denotes the start of subjective night in constant conditions, as evidenced by activity onset. Activity data were analyzed using ClockLab (Actimetrics Inc., United States), running within Matlab (Mathworks, United States). Circadian period (chi-squared periodogram analysis) and mean DD activity profiles were calculated for each animal, where activity was averaged over 8–10 days of activity and organized into 0.1 h bins.

### Implantation of EEG/EMG Transmitters

There were no significant differences in body weights at the time of surgery (WT = 30.5 ± 1.6g; *n* = 9; chimeric = 28.5 ± 1.1g; *n* = 8; *Tau* control = 29.3 ± 2.1g; *n* = 8). Mice were anaesthetized using isoflurane (induction 2–4%; maintenance 1%) with body temperature thermostatically controlled using a heating pad. Rimadyl was used for post-operative analgesia. Under aseptic conditions, animals were subcutaneously implanted with a telemetric transmitter (TL11M2-F20-EET, Data Sciences International, St Paul, MN, United States) connected to electrodes for continuous electroencephalography (EEG) and electromyography (EMG) recordings. Two screws were implanted above the dura (+1.5 mm anterior to Bregma and +1.7 mm lateral to Bregma, the second +1.0 mm anterior and +1.7 mm lateral to Lambda) around which the electrodes for measuring the EEG were placed and secured using dental cement (RelyX Unicem 2 automix; Henry Schein Animal Health, Dumfries, United Kingdom). The two EMG leads were inserted into the trapezius muscle ca 5 mm apart and sutured in place (Hasan et al., 2011).

### EEG/EMG Recordings

Following surgery, mice were allowed to recover for 2 weeks before the transmitters were activated on the day before data collection in both entrained (12L:12D and 10L:10D) and free-running conditions (DD). Mice were excluded from analysis where sleep-wake data contained excess artifacts (>10%). The EEG/EMG recordings were recorded continuously from the freely moving animals using Data Sciences International hardware and Dataquest ART v2.3 Gold software (Data Sciences International, ST Paul, MN, United States). EEG signals were low-pass filtered with a 30 Hz cut-off and collected simultaneously at a sampling rate of 500 Hz.

### Determination of Vigilance States and Spectral Analysis

Vigilance states for consecutive 4-s epochs were classified by visual inspection, and blind to genotype, according to standard criteria: wakefulness (high and variable EMG signal, low-amplitude EEG signal), NREM sleep (NREMS; high EEG amplitude dominated by slow waves, low EMG), and REM sleep (REMS; low EEG amplitude, theta oscillations and muscle atonia). Vigilance states were analyzed offline using Neuroscore Software (Data Sciences International) with the EEG and EMG signals modulated with a high-pass (3 dB, 0.5 Hz) and a low-pass (50 Hz) analog filter. For LD or DD conditions, continuous recordings were analyzed and time spent in each vigilance state was expressed as a percentage of the total recording time over various intervals (1–24 h). All DD recordings were started after at least 7 days of constant conditions. The mean number of individual bouts of vigilance states were grouped as a function of their duration (8–12, 16–28, 32–60, 64–124, 128–252, 256–508, 512–1020, >1024 s) per hour during LD or DD. The mean duration of NREMS bouts was compared between ZT6-12 on baseline day and following 6 h of sleep deprivation (SD).

EEG power spectra were computed for consecutive 4 s epochs over a circadian cycle by a fast-Fourier transform (frequency range: 0.5–49.80 Hz; resolution 0.24 Hz; Hanning window function). Genotypic differences were determined in DD over a complete circadian cycle and expressed as a percentage of total EEG power within each vigilance state for each mouse. The time course of spectral activity was also computed in 2 h bins during LD for delta (1–4 Hz) during NREMS and during/post 6 h SD and calculated as a percentage of the mean 24 h baseline for each mouse. Epochs containing EEG artifacts were discarded from the analysis.

### Sleep Deprivation

Mice were entrained to the 12L:12D photoschedule before recording sleep over a 24 h baseline day followed by 6 h SD and a further 18 h recovery sleep. SD (ZT0-6) involved gentle procedures, i.e., introduction of novel objects such as nesting material, “fun tubes” and an initial cage change.

### Novel Object Recognition Test

All experiments were performed in dim red light (5.5 ± 0.4 lux) between ZT20 and ZT22 under a 12L:12D photoschedule (Figure 1A). Experiments were performed in a red Perspex box measuring 50 × 50 × 50 cm with an overhead camera (Logitech Carl Zeiss Tessar HD 1080P) placed above the arena. The mice were habituated to the arena for 10 min, followed by an initial familiarization session 24 h later where they were exposed to two identical objects for 10 min (plain or patterned Perspex objects, e.g., square, pyramid, oval, egg-cup all of similar sizes). After 24 h, the mice were re-tested with one of the objects being replaced by a novel object of similar size. The percentage time the animals spent exploring each object in both the familiarization and test sessions were analyzed offline from the video recordings (using software designed by the laboratory of Prof W. Wisden, Imperial College, London, United Kingdom) (Yu et al., 2014) with the experimenter blind to the genotype of the animal.

### Organotypic Slices

Suprachiasmatic nucleus and other local brain tissue slice cultures were prepared as previously described (Maywood et al., 2010). Extra-SCN sites examined included regions reported to be involved in sleep/wake regulation, cognition and entrainment: preoptic area (POA), tuberomammillary nucleus (TMN), lateral hypothalamus (LH), and hippocampus (Hip). Slices were recorded from the day of preparation. Bioluminescence emissions from the whole slice were measured by photon multiplier tubes (Hamamatsu, Japan) (Maywood et al., 2006). All mice were housed for at least 7 days on 12L:12D prior to tissue collection (between ZT3-5) at the end of the experiment.

### Statistical Analysis

One or two-way ANOVA with *post hoc* Tukey’s or Sidak’s multiple comparisons were used to compare changes in sleep/wake parameters across genotypes (Prism version 8.0 for macOS X, GraphPad software). Comparisons of sleep–wake bouts, duration and frequency were made using a paired two-tailed Student’s *t*-tests within genotype and ANOVA between genotypes. Rayleigh tests for significant phase-clustering between *ex vivo* tissue explants were conducted in Oriana software (Kovach Computing Anglesey, United Kingdom), with the bioluminescence peak occurring between 12 and 36 h after culture as the phase reference.

## Results

### Circadian Wheel-Running Behavior in Chimeric Mice

Wild-types mice entrained stably to the 12L:12D cycle but not to 10L:10D, their activity scanning across the 10L:10D cycle and/or showing varying degrees of behavioral masking (Figure 1B). Conversely, *Tau* mice entrained stably to 10L:10D but not the 12L:12D cycle. Chimeric mice, however, exhibited stable activity rhythms to both the 12L:12D and the 10L:10D cycles. These effects are evident in the mean activity profiles, with significant light-phase activity in the *Tau* group in 12L:12D and the WT group in 10L:10D, respectively, due to their failure to entrain (Figure 1C). In contrast, chimeric mice did not show light-phase activity in either photoschedule. In DD, all groups showed significant circadian rhythmicity, confirming the robustness of the chimeric circadian system. The stable free-running periods were genotype-specific (Figure 1D), ca. 24 h for WT and 20 h for *Tau*, whilst wheel-running in chimeric mice had a period of 23.7 ± 0.4 h, i.e., a characteristic “Revertant” phenotype (Smyllie et al., 2016). The mean behavioral periods in DD for WT and chimeric mice were not significantly different but both were longer than *Tau* controls (one-way ANOVA: *F*_2,22_ = 150, *p* < 0.0001). These period differences were reflected by the phase angles of entrainment (Figure 1E). In 12L:12D this was not significantly different between WT and chimeric mice (WT = –0.1 ± 0.2 h; chimera = 0.2 ± 0.2 h; *n* = 6; *t* = 0.9, df 10, *p* = 0.4), whereas on 10L:10D there was a significant delay in activity onset in the chimeras compared to *Tau* mice (*Tau* = –0.1 ± 0.3 h; chimera = –1.2 ± 0.1 h, *n* = 6–7; *t* = 3.2, df 11, *p* < 0.01) (n.b. the inability of *Tau* controls and WT to entrain 12L:12D and 10L:10D, respectively, precluded their inclusion in this analysis). The *Drd1a*-mediated deletion of the *CK1e*^*Tau*^ alleles therefore generated temporally chimeric mice, with a stable ca. 24 h period but with an ability to entrain to both 12L:12D and 10L:10D cycles.

### Bioluminescence Rhythms in the Brain of Chimeric Mice

**FIGURE 2:**
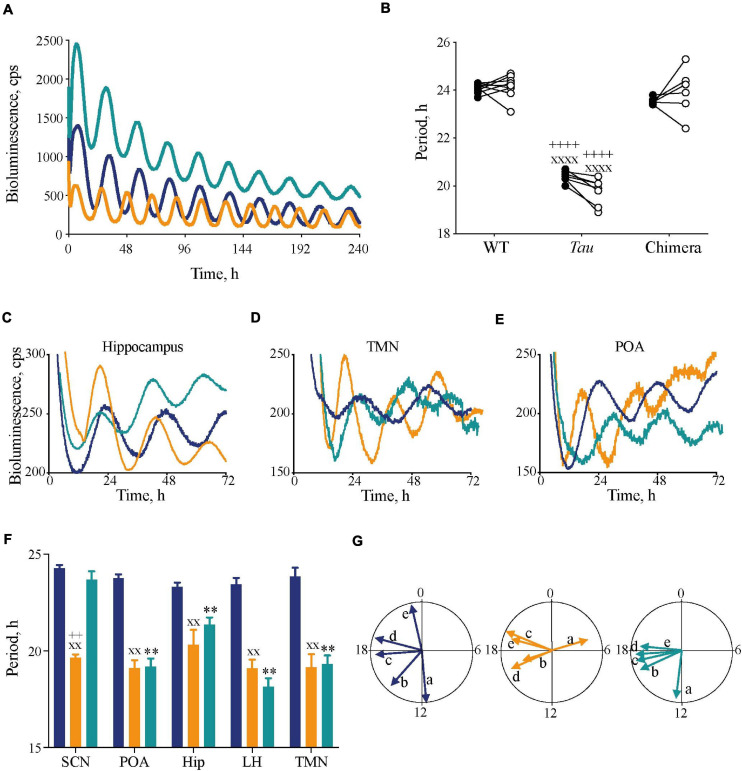
Impact of circadian chimerism on expression of bioluminescence rhythms of PER2::Luc in SCN and extra-SCN tissues. **(A)** Representative PER2::Luc bioluminescence traces from *ex vivo* SCN slices from WT (blue), *Tau* (orange), and chimeric (green) mice. **(B)** Individual circadian periods for wheel-running in DD (closed circles) and PER2::Luc-reported SCN bioluminescence rhythms (open circles) (*n* = 6–9). Note the effect of genotype within, but not between, *in vivo* and *ex vivo* measures (1xANOVA *post hoc* Tukey’s multiple comparisons test xxxx*p* < 0.0001 *Tau* vs. WT, ++++*p* < 0.0001 *Tau* vs. chimera). **(C–E)** Representative traces of PER2::Luc bioluminescence rhythms from *ex vivo* hippocampus **(C)**, tuberomammilary nucleus (TMN) **(D)**, and preoptic area (POA) **(E)** tissue slices from WT (blue), *Tau* (orange), and chimeric (green) mice. **(F)** Mean (±SEM) period (*n* = 5–9) of *ex vivo* tissue slices from WT (blue), *Tau* (orange), and chimeric (green) mice. All tissues, except SCN, from chimeric mice have a *Tau*-characteristic period of ca. 20 h (two-way ANOVA *post hoc* Tukey’s multiple comparisons test; ^∗∗^*p* < 0.01 WT vs. chimera, xx*p* < 0.01 *Tau* vs. WT, ++*p* < 0.0001 *Tau* vs. chimera). **(G)** Rayleigh plots of the mean vector for all tissue explants from WT (blue), *Tau* (orange), and chimeric (green) mice (see also Table 1). Note the phase-clustering in both *Tau* and chimeric, but not WT tissues (Rayleigh test). [a = SCN; b = Preoptic area; c = Hippocampus (Hip); d = tuberomammilary area; e = lateral hypothalamus (LH)].

To characterize temporal chimerism across the brain, circadian rhythms of bioluminescence were measured *ex vivo* at study completion from organotypic slices of the SCN and other brain regions implicated in the regulation of sleep/wake cycles and cognition: POA, LH, TMN, and Hip. WT and *Tau* control SCN showed intrinsic periods of ca. 24 and 20 h respectively (Figures 2A,B). The SCN from chimeric mice oscillated at ca. 24 h confirming the efficacy of local *CK1*ε*^*Tau/Tau*^* deletion by *Drd1a*-driven recombinase. Importantly, in all three groups the *ex vivo* SCN period was not significantly different from the behavioral period of the mouse (two-way ANOVA: Interaction *F*_2,38_ = 4.0, *p* < 0.05; Genotype *F*_2,38_ = 303, *p* < 0.0001; Period *F*_1,38_ = 0.13, *p* = 0.7) (Figure 2B). The contrasting behavioral periods could therefore be ascribed to the intrinsic periods of the SCN.

The circadian periods of the other brain tissue explants from WT and *Tau* control mice were also genotype-specific, at ca. 24 and 20 h, consistent with their respective SCN clock (Figures 2C–F). The non-SCN tissues from the chimeras, however, showed clear ca. 20 h periods, significantly shorter than the corresponding SCN (chimera: one-way ANOVA *F*_4,25_ = 29, *p* < 0.0001; Tukey’s multiple comparisons test *p* < 0.0001 SCN vs. LH, POA, and TMN; *p* < 0.05 SCN vs. Hip). Temporal chimerism was also reflected in the relative phasing of the molecular oscillations observed in different brain areas (Figure 2G). Across all genotypes, tissue slices showed significant phase-clustering (*p* < 0.05, Rayleigh test) apart from the *Tau* control POA (Table 1). The bioluminescence oscillations of SCN from WT and chimeric mice peaked around circadian time CT12, as defined by their prior behavior. This was more variable in the SCN from *Tau* mice (mean ± SD; CT4.9 ± 3.1 h), reflecting their imprecise entrainment to 12L:12D. As anticipated, the tissues from other brain regions of WT mice exhibited a broad range of phases (range: 2–12 h; Figure 2G and Table 1) relative to the corresponding SCN (Yoo et al., 2005; Maywood et al., 2010). Conversely other brain tissues from the *Tau* and chimeric mice had peak phases within ca. 3 h of each other. This altered internal synchronization between brain areas likely reflects the disrupted entrainment of *Tau* control mice to LD, and temporal chimerism in the behaviorally 24 h chimeric mice. Thus, targetted deletion of the *CK1*ε*^*Tau/Tau*^* allele created mice in which cell-autonomous periods were different between SCN and local clocks and in which *ex vivo* recordings indicated correspondingly altered internal phasing.

**TABLE 1:** Mean circadian phase and vector of *ex vivo* tissues from mice (Related to Figure 2).

Genotype Tissue	WT		Chimera		*Tau*	
	Mean time (n ± sd)	Mean vector	Mean time (n ± sd)	Mean vector	Mean time (n ± sd)	Mean vector
SCN	11.69 ± 1.22 *n* = 9	0.95 (*p* < 0.05)	12.42 ± 1.42 *n* = 8	0.93 (*p* < 0.05)	4.9 ± 3.06 *n* = 7	0.68 (*p* < 0.05)
Preoptic area (POA)	14.66 ± 1.71 *n* = 6	0.90 (*p* < 0.05)	16.33 ± 2.01 *n* = 7	0.86 (*p* < 0.05)	16.72 ± 3.51 *n* = 7	0.58 (ns)
Hippocampus (Hip)	17.73 ± 2.2 *n* = 7	0.83 (*p* < 0.05)	17.16 ± 2.1 *n* = 5	0.85 (*p* < 0.05)	19.54 ± 0.57 *n* = 5	0.99 (*p* < 0.05)
Lateral hypothalamus (LH)	19.02 ± 1.78 *n* = 9	0.89 (*p* < 0.05)	17.74 ± 2.18 *n* = 6	0.84 (*p* < 0.05)	16.71 ± 2.03 *n* = 7	0.86 (*p* < 0.05)
Tuberomammilary nucleus (TMN)	23.18 ± 1.22 *n* = 5	0.95 (*p* < 0.05)	18.32 ± 2.68 *n* = 5	0.75 (*p* < 0.05)	19.03 ± 2.45 *n* = 7	0.79 (*p* < 0.05)

### Altered Sleep–Wake Architecture in Circadian Chimeric Mice

**FIGURE 3:**
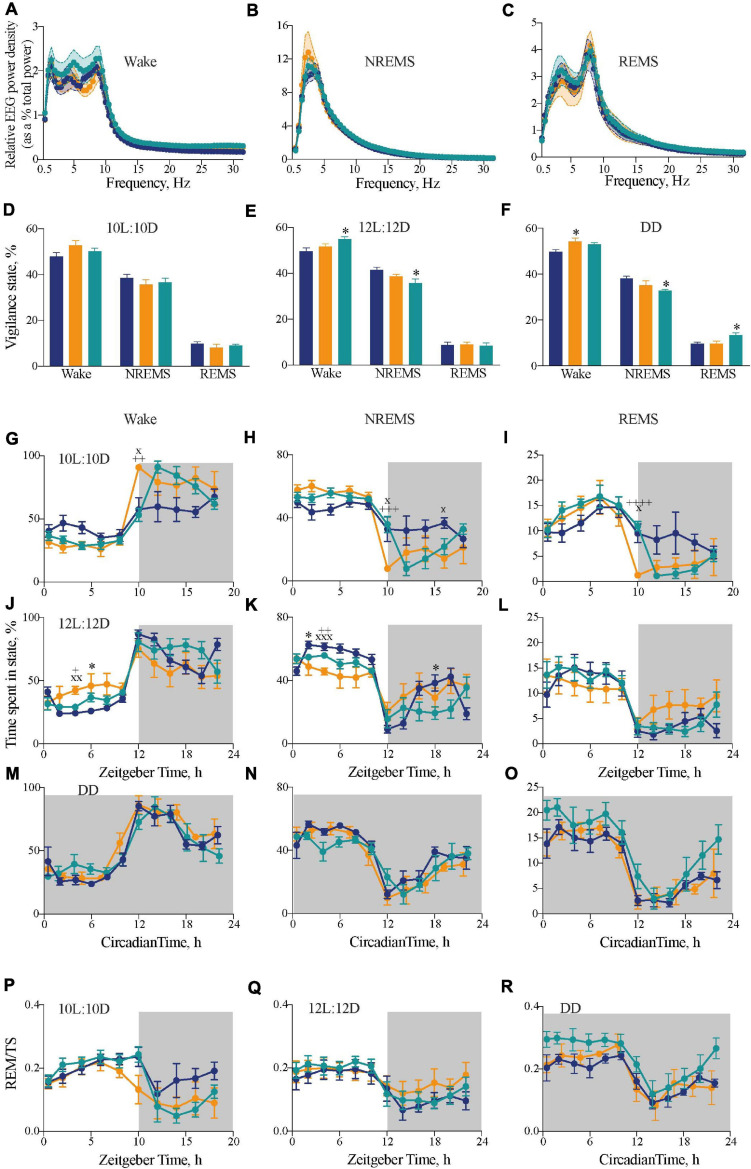
Impact of circadian chimerism on sleep-wake cycles. **(A–C)** Mean (±SEM) relative EEG spectral power (as a percentage of total EEG power) across 0–30 Hz in wake **(A)**, NREMS **(B)**, and REMS **(C)** over a circadian cycle. WT (blue), *Tau* (orange), and chimeric (green) mice (two-way ANOVA with *post hoc* Tukey’s multiple comparisons test; no significant effects between Genotype or an Interaction between Genotype × Frequency in any vigilance state; *n* = 5–6). **(D–F)** Distribution of percentage time (mean ± SEM) spent in each vigilance state of wake, NREM and REM sleep under entraining conditions of 10L:10D **(A)**, 12L:12D **(B)**, and free-running conditions in DD **(C)**. WT (blue; *n* = 6–9), *Tau* (orange; *n* = 5–7), and chimeric (green; *n* = 6–7) mice (two-way ANOVA with *post hoc* Tukey’s multiple comparisons test, ^∗^*p* < 0.05 WT vs. chimera; WT vs. *Tau*). **(G–L)** Two hourly distribution (mean ± SEM) of percentage time spent in wake **(G,J)**, NREM **(H,K)**, and REM **(I,L)** sleep under entraining conditions of 10L:10D **(G–I)** or 12L:12D **(J–L)**. **(M–O)** Distribution of percentage time (mean ± SEM) spent in wake **(M)**, NREM **(N)**, and REM **(O)** sleep aligned to circadian time 12 (CT12; activity onset) under constant DD conditions. **(P–R)** Distribution of percentage time (mean ± SEM) spent in REMS as a percentage of total sleep (TS = NREMS + REMS) in either 10L:10D **(P)**, 12L:12 **(Q)**, or DD **(R)**. [WT (blue; *n* = 6–9), *Tau* (orange; *n* = 5–7), and chimeric (green; *n* = 6–7) mice]. (2x ANOVA with *post hoc* Tukey’s multiple comparisons test, ^∗^*p* < 0.05, WT vs. chimera; x*p* < 0.05, xx*p* < 0.01, xxx*p* < 0.001 WT vs. *Tau*; +*p* < 0.05, ++*p* < 0.01, +++*p* < 0.005, ++++*p* < 0.0001 chimera vs. *Tau*).

The chimeric mice therefore had a dominant SCN-determined 24 h behavioral period, set against 20 h local clocks and altered internal phasing. Did this discordance between SCN and local clocks affect the timing and/or quality of sleep? To confirm that the genotypes did not affect the signature spectra of wake, NREMS and REMS vigilance states, EEG/EMG recordings were analyzed from freely moving mice under DD conditions, where there were no confounding issues with light exposure on vigilance (Figures 3A–C). For all three vigilance states, there were no significant differences between the three groups in the EEG power density between 0.5 and 30 Hz, confirming that temporal chimerism did not alter the characteristic EEG oscillations of sleep and wake (two-way RM ANOVA Wake: Interaction *F*_124,806_ = 0.5 ns; Frequency *F*_2.7,35_ = 145, *p* < 0.0001; Genotype *F*_2,13_ = 1.4 ns; NREMS: Interaction *F*_124,806_ = 0.7 ns; Frequency *F*_1.5,19_ = 198, *p* < 0.0001; Genotype *F*_2,13_ = 0.2 ns; REMS: Interaction *F*_124,806_ = 1.1 ns; Frequency *F*_2.8,37_ = 352, *p* < 0.0001; Genotype *F*_2,13_ = 0.9 ns). We then examined the amount of time spent in each state across the entire 20 h, 24 h, and circadian cycles. Under 10L:10D, there were no significant differences between genotypes (Figure 3D), whereas over a 12L:12D cycle there was a significant increase in wakefulness with concomitant reduction (ca 80 min) in NREMS in the chimeric mice compared to WT controls (one-way ANOVA: Wake: *F*_2,20_ = 4.2, *p* < 0.05; Tukey’s multiple comparisons test *p* < 0.05; NREMS: *F*_2,20_ = 4.9, *p* < 0.05; Tukey’s multiple comparisons test *p* < 0.05) (Figure 3E). Importantly, in the absence of any effects of light under DD (Figure 3F), chimeric mice also showed a significant reduction in NREMS and concomitant increase in REMS duration compared to WTs (one-way ANOVA: NREMS: *F*_2,14_ = 4.1, *p* < 0.05; Tukey’s multiple comparisons test *p* < 0.05; REMS: *F*_2,14_ = 4.2, *p* < 0.05; Tukey’s multiple comparisons test *p* < 0.05).

The distributions of sleep and wake across the 20 h, 24 h, and circadian cycles varied between groups. Under 10L:10D, WT mice did not entrain and so their sleep/wake spread equally across the 20 h cycle. In contrast, both *Tau* and chimeric mice entrained with the expected distribution of sleep in the light phase and nocturnal wakefulness (Figures 3G–I) (two-way RM ANOVA Wake: Interaction *F*_18,126_ = 3.6, *p* < 0.001; Time *F*_4,60_ = 30.1, *p* < 0.0001; Genotype *F*_2,14_ = 1.6 ns; NREMS: Interaction *F*_18,126_ = 3.5 *p* < 0.0001; Time *F*_4,58_ = 29.6, *p* < 0.0001; Genotype *F*_2,14_ = 0.6 ns; REMS: Interaction *F*_18,126_ = 3.1, *p* < 0.0001; Time *F*_3,36_ = 24.2, *p* < 0.0001; Genotype *F*_2,14_ = 0.6 ns). In this 20 h cycle, however, the chimeric mice showed a significant delay in the transition from sleep to wake at the light to dark transition compared with the perfectly entrained *Tau* mice. This is consistent with the delayed onset of wheel-running behavior seen in chimeras under 10L:10D (Figure 1D), and suggests it is caused by a delay in waking rather than a delay specifically in wheel-running locomotor activity.

In 12L:12D, the poorly entrained *Tau* mice showed poor segregation of sleep and wake across day and night, whereas WT and chimeric mice showed the characteristic peak of wake at the onset of darkness (Figures 3J–L). Nevertheless, the chimeric mice did not show the typical “siesta” during the dark phase compared to the WT mice. Thus, despite showing stably entrained behavior to the 24 h cycle, the temporal structure of sleep/wake was significantly altered in the circadian chimeras (two-way RM ANOVA Wake: Interaction *F*_22,220_ = 2.5, *p* < 0.0005; Time *F*_4,79_ = 28.1, *p* < 0.0001; Genotype *F*_2,20_ = 1.1 ns; NREMS: Interaction *F*_22,220_ = 2.9, *p* < 0.0001; Time *F*_4,79_ = 25.5, *p* < 0.0001; Genotype *F*_2,20_ = 2.8 ns; REMS: Interaction *F*_22,220_ = 1.6, *p* < 0.05; Time *F*_3,69_ = 21.2, *p* < 0.0001; Genotype *F*_2,20_ = 0.2 ns).

The sleep–wake time course of all three genotypes under DD showed significant organization of wake, NREMS and REMS across the circadian cycle when plotted in circadian time and aligned to CT12 (Figures 3M–O) (1xANOVA Wake: WT: *F*_11,60_ = 15.9, *p* < 0.0001; Tau: *F*_9,40_ = 11.2, *p* < 0.0001; Chimera: *F*_11,60_ = 8.3, *p* < 0.0001: NREMS: WT: *F*_11,60_ = 11.0, *p* < 0.0001; *Tau*: *F*_9,40_ = 8.9, *p* < 0.0001; Chimera: *F*_11,60_ = 6.1, *p* < 0.0001; REMS: WT: *F*_11,60_ = 11.6, *p* < 0.0001; *Tau*: *F*_9,40_ = 6.8, *p* < 0.0001; Chimera: *F*_11,60_ = 6.8, *p* < 0.0001). Furthermore, analysis of REMS as a proportion of total sleep (TS = NREMS + REMS), a measure independent of changes in the overall amount of sleep, was rhythmic in all three genotypes under both entrained and free-running conditions (Figures 3P–R), with significantly more REMS/TS in the chimeric *versus* WT animals over the entire cycle in DD (unpaired *t*-test: *t* = 2.8, df = 10, *p* < 0.02). Thus, even though circadian control was intact, the level of REMS was proportionately greater in chimeras. Overall, under 12L:12D and DD, the main differences between WT and chimeras was a ca. 80 min decrease in the amount of NREM sleep (Figures 3E,F), which was distributed across the cycle, and a delayed transition from sleep to wake in 10L:10D (Figures 3G–I). This suggests a role for the SCN in maintaining sleep/suppressing wake toward the end of the light period, and so delaying the switch from rest to active wake in the chimeras. Together, the results highlight the plasticity of the SCN in driving both locomotor activity and regulating sleep–wake duration and temporal distribution, and suggest a coherent signal from the *Drd1a*-expressing cells in the SCN is necessary for the switch between NREMS to wake.

### Fragmented Sleep in Chimeric Mice

**FIGURE 4:**
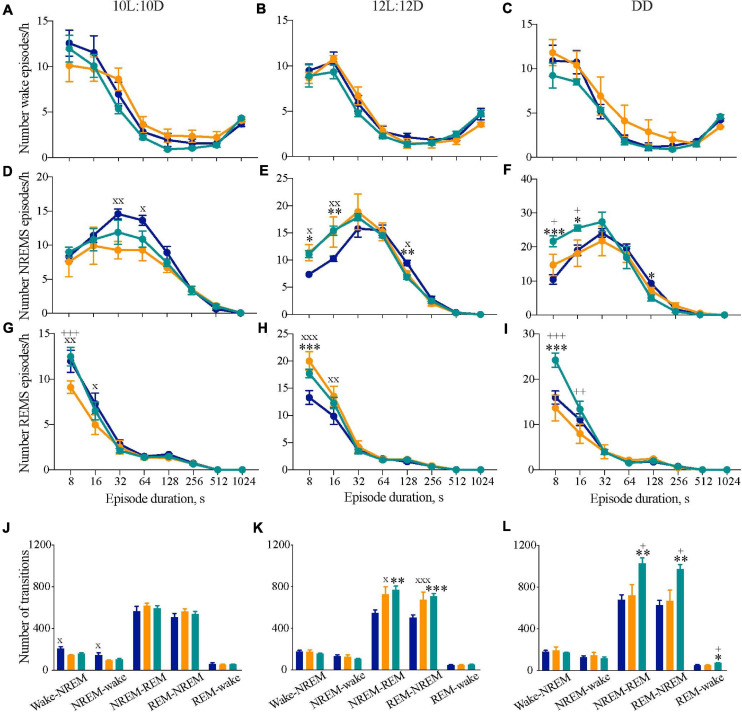
Disrupted sleep continuity in circadian chimeric mice. **(A–I)** Frequency distribution (mean ± SEM) of the mean number of episodes per bin (expressed per hour of its respective vigilance state) of wakefulness **(A–C)**, NREM sleep **(D–F)**, and REM sleep **(G–I)** throughout consecutive time bins (8–12, 16–28, 32–60, 64–124, 128–252, 256–508, 512–1020, >1024 s) over the entraining 10L:10D cycle **(A,D,E)**, 12L:12D cycle **(B,E,H)** and under free-running DD **(C,F,I)** in WT (blue, *n* = 6–9), *Tau* (orange, *n* = 5–7), and chimeric mice (green, *n* = 6–7). Values are plotted against the lower limit of each bin. **(J–L)** Mean number of transitions between vigilance states under entraining 10L:10D cycle **(J)**, 12L:12D cycle **(K)**, and free-running DD **(L)** in WT (*n* = 6–9, blue), *Tau* (*n* = 5–7, orange), and chimeric (*n* = 6–7, green) mice (1xANOVA with *post hoc* Tukey’s multiple comparisons test, ^∗^*p* < 0.05; ^∗∗^*p* < 0.01; ^∗∗∗^*p* < 0.0001 WT vs. chimera; +*p* < 0.05, ++*p* < 0.01, +++*p* < 0.001 chimera vs. *Tau*; x*p* < 0.05, xx*p* < 0.01, xxx*p* < 0.001 WT vs. *Tau*).

We hypothesized that circadian mis-alignment may result in increasedfragmentation of sleep. The distribution of the frequency of NREM andREM sleep episodes throughout eight consecutive time bins for10L:10D, 12L:12D, or DD revealed differences in NREMS and REMS between the genotypes (Figure 4). In 10L:10D there were differences between WT and *Tau* mice in NREMS and REMS, and differences between the well entrained *Tau* and chimeric mice in REMS but not NREMS or wake (Figures 4A,D,G) (two-way RM ANOVA: NREMS: Interaction *F*_14,105_ = 1.4 ns; Duration *F*_7,105_ = 74.4, *p* < 0.0001; Genotype *F*_2,15_ = 1.7 ns; REMS: Interaction *F*_14,105_ = 2.0, *p* < 0.05; Duration *F*_7,105_ = 177.3, *p* < 0.0001; Genotype *F*_2,15_ = 1.8 ns; Tukey’s multiple comparisons test *p* < 0.05, 0.01 WT vs. *Tau*; *p* < 0.001 *Tau* vs. chimera). Under 12L:12D both the *Tau* and chimeric animals had significantly more of the shorter NREMS and REMS bouts than WT animals (Figures 4E,H), suggesting a more fragmented sleep in these mice that either entrained (chimeras) or did not (*Tau*) to the light:dark photoschedule (two-way RM ANOVA: NREMS: Interaction *F*_14,112_ = 2.5, *p* < 0.005; Duration *F*_7,112_ = 150.3, *p* < 0.0001; Genotype *F*_2,16_ = 1.0 ns; REMS: Interaction *F*_14,112_ = 3.8, *p* < 0.0001; Duration *F*_7,112_ = 272.9, *p* < 0.0001; Genotype *F*_2,16_ = 2.4 ns; Tukey’s multiple comparisons test *p* < 0.001 chimera vs. WT; *p* < 0.05, *p* < 0.01, *p* < 0.0001 *Tau* vs. WT). The significantly higher frequency of shorter bout lengths in the chimeras compared with WT mice was also evident in DD for both NREMS and REMS (Figures 4F,I) (two-way RM ANOVA: NREMS: Interaction *F*_14,98_ = 3.3, *p* < 0.001; Duration *F*_7,98_ = 117.6, *p* < 0.0001; Genotype *F*_2,14_ = 1.1 ns; REMS: Interaction *F*_14,98_ = 6.1, *p* < 0.0001; Duration *F*_7,98_ = 170.9, *p* < 0.0001; Genotype *F*_2,14_ = 2.9 ns; Tukey’s multiple comparisons test *p* < 0.001 chimera vs. WT; *p* < 0.001 *Tau* vs. chimera). Importantly, under DD the *Tau* mice were comparable to WT. Thus, when the circadian system is free-running, in the temporally misaligned chimeric mice sleep is fragmented compared to temporally coherent WT and *Tau* mice.

We also studied the number of transitions between vigilance states as another index of sleep–wake architecture. While there were no significant differences in the number of NREMS-REMS transitions when the animals were housed in 10L:10D, there was a significant difference in the number of wake-NREMS-wake transitions in WT mice compared to *Tau* mice (1xANOVA Wake-NREMS: *F*_2,15_ = 6.2, *p* < 0.01; NREMS-wake: *F*_2,15_ = 4.0, *p* < 0.05; Tukey’s multiple comparisons test WT vs. *Tau p* < 0.05). In 12L:12D, however, both chimeras and *Tau* animals had more transitions between NREMS-REMS and REMS-NREMS compared with WT mice, consistent with the higher frequency of shorter episodes (Figures 4E,H,K) (1xANOVA NREMS-REMS: *F*_2,20_ = 6.3, *p* < 0.01; REMS-NREMS: *F*_2,20_ = 6.5, *p* < 0.01; Tukey’s multiple comparisons test WT vs. chimera *p* < 0.01, WT vs. *Tau p* < 0.05). Under DD, fragmentation was again evident in chimeras but not, however, in *Tau* mice (Figure 4L) (NREMS-REMS: *F*_2,16_ = 7.1, *p* < 0.01; REMS-NREMS: *F*_2,16_ = 7.8, *p* < 0.005; Tukey’s multiple comparisons test WT vs. chimera *p* < 0.01, chimera vs. *Tau p* < 0.05). Fragmentation could not, therefore, be ascribed to the *Tau* mutant background of the chimeras. The circadian chimerism was accompanied by some fragmentation of the sleep–wake cycle under entrained conditions, but was even more evident when the clocks were free-running under DD conditions. Therefore, circadian mis-alignment between the SCN and extra-SCN clocks, but not WT or *Tau* genetic background, significantly impaired sleep consolidation in the chimeras.

### Effect of Enhanced Sleep Pressure in Circadian Chimeras

**FIGURE 5:**
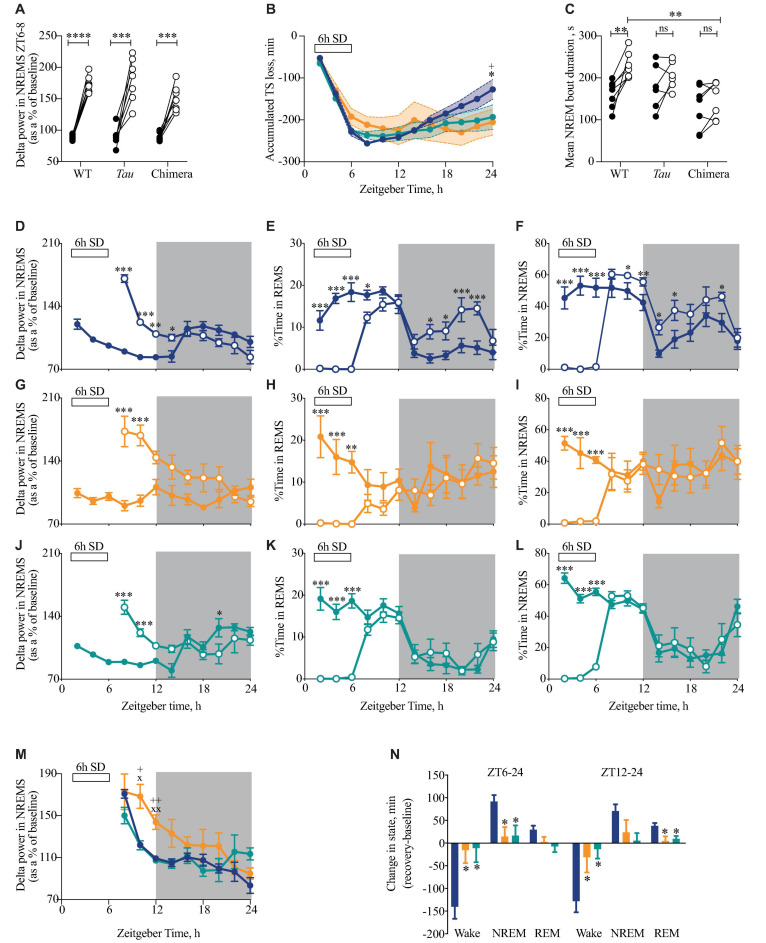
Attenuated response to sleep deprivation (SD) in circadian chimeric mice. **(A)** Individual changes in EEG delta power in NREMS (1–4 Hz) (as a percentage of total power in baseline day) in the first 2 h of recovery sleep (ZT6-8; open circles) compared with baseline sleep (ZT6-8; closed circles) in WT, *Tau* and chimeric mice (two-tailed paired *t*-test show baseline vs. post-SD within genotype; ^***^p < 0.01, ^****^p < 0.0001). **(B)** The cumulative loss of total sleep (TS = NREMS + REMS) over the 6 h of SD with subsequent partial recovery of TS during the 18 h recovery period (ZT30-48) in WT (blue; *n* = 9), *Tau* (orange; *n* = 7), and chimeric (green; *n* = 7) mice (2xANOVA with post hoc Tukey’s multiple comparisons test *p* < 0.05, ^∗^WT vs. chimera; +WT vs. *Tau*). **(C)** Individual changes in the duration of bouts of NREMS (seconds) between ZT6-12 on baseline day (closed circles) and after 6 h SD (open circles) (Paired *t*-tests show baseline vs. 6 h SD difference within genotype ^∗∗^*p* < 0.01; 1xANOVA with *post hoc* Tukey’s multiple comparisons test ^∗∗^*p* < 0.01 WT vs. chimera). **(D–L)** Time course at 2 h intervals of delta power (mean ± SEM) in NREMS as a percentage of the total power during baseline **(D,G,J)**, REMS **(E,H,K)**, NREMS **(F,I,L)** in WT (blue; **D–F**), *Tau* (orange; **G–I**), and chimeric mice (green; **J–L**) on the baseline day (closed circles) and following 6 h SD (open circles). (2xANOVA with *post hoc* Tukey’s multiple comparison test (REMS, NREMS) or Sidak’s multiple comparisons test (delta power in NREMS) ^∗∗∗^*p* < 0.0001; ^∗∗^*p* < 0.001, ^∗^*p* < 0.01 vs. baseline day). **(M)** Time course at 2 h intervals of delta power (mean ± SEM) in NREMS (as a percentage of baseline) following 6 h SD in WT (blue), *Tau* (orange), and chimeric mice (green). (2xANOVA with *post hoc* Sidak’s multiple comparisons test x*p* < 0.05, xx*p* < 0.01 WT vs. Tau; +*p* < 0.05, ++*p* < 0.01 *Tau* vs. chimera) (same data as plotted in D,G,J). **(N)** Differences between the change in state (mean ± SEM) for wake, NREMS and REMS following 6 h SD compared with baseline between ZT6-24 (light and dark phase of recovery) and ZT12-24 (dark phase only) in WT (blue), *Tau* (orange), and chimeric mice (green) (1xANOVA with Tukey’s multiple comparisons test *p* < 0.05 vs. WT; *n* = 7–9).

Having demonstrated that circadian misalignment results in a decrease in the daily amount of NREMS and a fragmentation of sleep, we next tested whether this has any impact on the homeostatic response to 6 h of sleep loss (Figure 5). During SD, chimeric mice were more difficult to keep awake, with a significantly higher amount of NREM sleep during the last 2 h of SD (WT = 3.6 ± 1.4 min, chimeras = 10.6 ± 2.7 min, *Tau* = 4.4 ± 1.9 min; one-way ANOVA *F*_2,20_ = 3.7, *p* < 0.05; *post hoc* Tukey’s multiple comparisons test WT vs. chimera *p* < 0.05). Despite chimeras being more difficult to keep awake, there were no significant differences between WT and chimeric mice (or *Tau* mice) in the initial response to SD, as assessed by measuring the EEG delta power in NREMS (between 1 and 4 Hz) in the 2 h immediately following 6 h SD (ZT6-8) compared to the same time on the baseline day (Figure 5A) (2xANOVA: Interaction *F*_2,20_ = 2.3 ns; genotype *F*_2,20_ = 3.0 ns; SD *F*_1,20_ = 198, *p* < 0.0001). The chimeric condition did not, therefore, compromise their ability to compensate for sleep deprivation and generate the appropriate neurophysiological response to it when NREMS occurred.

The time course of recovered sleep was, however, significantly altered in chimeric compared to WT mice over the 18 h recovery period (Figure 5B). Whereas sleep debt was progressively paid off in WT mice, the accumulated recovery of sleep loss between ZT6-24 following SD was significantly altered in the chimeras and *Tau* mice (2xANOVA Interaction: *F*_22,231_ = 3.0, *p* < 0.0001; Time: *F*_11,231_ = 47.2, *p* < 0.0001; Genotype: *F*_2,21_ = 0.3 ns). This difference in recovery was also reflected in the duration of bouts of NREMS over the 6h immediately following SD (ZT6-12). Whereas the WT mice showed significant increases relative to the prior baseline, this was not systematically the case for the chimeric mice, nor for the *Tau* mice (Figure 5C) (2xANOVA Interaction: *F*_2,17_ = 3.0 ns; Genotype *F*_2,17_ = 4.6, *p* < 0.05; SD *F*_1,17_ = 19.5, *p* < 0.005). This likely reflects a decreased sleep pressure and/or inability to maintain consolidated NREMS at this phase of the 12L:12D cycle. The lower response in terms of sleep recovery in *Tau* mice likely reflects their poor entrainment to 12L:12D, but this does not explain the poor response of the well entrained chimeras.

Changes in EEG delta power in NREMS (1–4 Hz) provides an additional index of the recovery process after 6 h SD. WT and chimeric animals showed the expected changes over the baseline day (Figures 5D,J), i.e., a decline in NREMS EEG delta power as sleep need was satiated during the light phase, whereas the un-entrained *Tau* mice did not show such a variation across the 12L:12D cycle (Figure 5G). Although there was a highly significant increase following the 6h of SD in all three groups (2xANOVA: WT: Interaction *F*_8,137_ = 23.2, *p* < 0.0001; Time: *F*_8,137_ = 12.1, *p* < 0.0001; SD: *F*_1,137_ = 27.6, *p* < 0.0001; *Tau*: Interaction *F*_8,91_ = 5.5, *p* < 0.0001; Time: *F*_8,91_ = 3.2, *p* < 0.005; SD: *F*_1,91_ = 46.7, *p* < 0.0001; Chimera: Interaction *F*_8,85_ = 9.6 *p* < 0.0001; Time: *F*_8,85_ = 5.2, *p* < 0.0001; SD: *F*_1,85_ = 6.5, *p* < 0.05), direct comparison revealed the slower recovery, as reported by delta power during NREMS, in un-entrained *Tau* mice (Figure 5M).

Beyond delta power, in the subsequent dark phase, sleep recovery continued in WT mice, which showed more time in NREMS and REMS than on the baseline day (Figures 5E,F). *Tau* mice did not show this additional recovery sleep (Figures 5H,I), consistent with their poor entrainment on 12L:12D and subsequently disorganized sleep–wake pattern. More importantly, the chimeras did not show this extended time in recovery sleep (Figures 5K,L) (2xANOVA REMS: Interaction: *F*_22,216_ = 3.3, *p* < 0.0001; Time: *F*_11,216_ = 16, *p* < 0.0001; genotype: *F*_2,216_ = 4.5, *p* < 0.05: NREMS: Interaction: *F*_22,216_ = 3.1, *p* < 0.0001; Time: *F*_11,216_ = 26.6, *p* < 0.0001; genotype: *F*_2,216_ = 6.5, *p* < 0.005). Consequently, significantly less NREMS was recovered in chimeric (and *Tau*) animals between ZT6-24 (Figure 5N) (1xANOVA Wake: *F*_2,20_ = 7.1, *p* < 0.005; NREMS: *F*_2,20_ = 5.9, *p* < 0.01). Equally, REMS was recovered predominantly during the dark phase (ZT12-24) in the WT but not chimeric (or *Tau*) mice (1xANOVA REMS: *F*_2,20_ = 6.3, *p* < 0.01). These results reveal that the chimeras have a reduced recovery sleep in response to SD over the subsequent 18 h time course, even though the neurophysiological mechanisms that enhance NREMS delta power were intact. The lack of significant differences between the (entrained) chimeric and the (non-entrained) *Tau* mice in the recovery of sleep loss confirms that recovery from enhanced sleep pressure can be affected by poor entrainment to the 12L:12D cycle (*Tau* mice), but they also reveal the effect of incoherence between brain regions in otherwise well entrained mice (chimeras).

### Effect of Circadian Chimerism on Memory Performance

**FIGURE 6:**
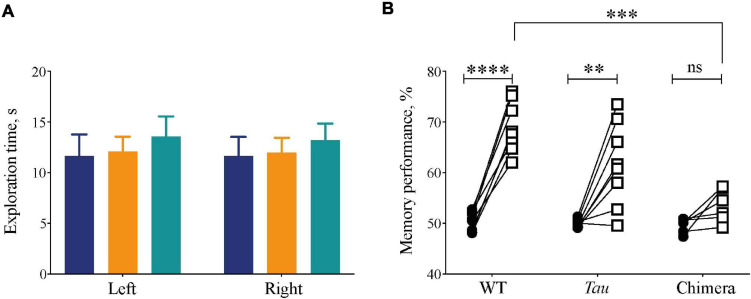
Compromised recognition memory performance in circadian chimeric mice. **(A)** The amount of exploration time (mean ± SEM) mice spent on the objects during the training session (identical objects) was not significantly different between groups (WT (blue), *Tau* control (orange), and chimeric (green) mice). **(B)** The percentage time (mean ± SEM) the mice spent on the novel object (open squares) was significantly greater than during the training phase (closed circles) in the WT and *Tau* mice, whereas chimeric mice performed poorly on the test phase (two-tailed paired Student’s *t*-test within genotype, ^∗∗^*p* < 0.01, ^****^p < 0.0001; 2x ANOVA between genotypes; *post hoc* Tukey’s multiple comparisons test ^∗∗∗^*p* < 0.001 WT vs. chimeras).

Internal circadian desynchrony in chimeric mice compromised sleep architecture, continuity and homeostasis. To determine whether this had functional consequences we used the Novel Object Recognition (NOR) task as an index of sleep-dependent memory (Palchykova et al., 2006). Mice trained with two identical objects during the dark phase of the 12L:12D cycle (after > 14 days) showed no genotype differences in the time spent exploring them (2xANOVA: Interaction: *F*_2,38_ = 0.006, *p* = 0.99; Genotype: *F*_2,38_ = 0.01, *p* = 0.91; Object: *F*_1,38_ = 0.4, *p* = 0.63) (Figure 6A). During the test 24 h later, however, whereas WT and *Tau* mice exhibited robust recognition memory, the chimeric mice failed to discriminate between the novel and the familiar objects, spending less time exploring the novel object than did WT and *Tau* controls (Figure 6B) (two-tailed Student’s paired *t*-test: WT: *t* = 11.5 df = 7, *p* < 0.0001; *Tau*: *t* = 4.1 df = 7, *p* < 0.004; chimera: *t* = 2.6 df = 5 ns; 2xANOVA: Interaction *F*_2,19_ = 10, *p* < 0.001; Genotype: *F*_2,19_ = 10, *p* < 0.001; Object: *F*_1,19_ = 80, *p* < 0.0001; Tukey’s multiple comparisons test WT vs. Chimera *p* < 0.001). Thus, circadian chimerism, rather than *Tau* genotypic background, compromised performance in a form of memory known to be sleep-dependent. Together, these results demonstrate that the effective organization of sleep and thereby sleep-dependent memory requires temporal coherence between the SCN and local extra-SCN circadian clocks.

## Discussion

Rapid progress has been made recently in mapping the neural circuits that direct states of sleep and wakefulness. In contrast little attention has been given to the temporal regulation of these pathways. Indeed, in contrast to the substantial advances made in understanding the contributions of peripheral circadian clocks to metabolic health, the roles of local circadian time-keeping across the brain remains relatively unexplored. To dissect the contribution of non-SCN circadian clocks to the regulation of sleep, we used temporally chimeric mice with 24 h clocks in the SCN but 20 h clocks elsewhere. Our finding that switching between sleep states, duration, consolidation and homeostasis, as well as sleep-dependent memory are compromised in circadian chimeric mice refutes the null hypothesis that extra-SCN clocks make no contribution to the control of sleep. Thus, notwithstanding the role of the SCN as the primary circadian oscillator, the circadian control of sleep is further distributed across the brain.

Chimeric mice exhibited 24 h behavioral cycles and SCN PER2::LUC rhythms, whereas other sleep-relevant brain sites expressed ca. 20h cell-autonomous circadian periods *ex vivo*. In the absence of *in vivo* imaging of tissue-specific oscillations in live mice (Curie et al., 2015; Hamada et al., 2016), we anticipate several consequences of chimerism. First, the 20 h clocks of local brain areas would be unable to adapt to SCN-determined 24 h cycles, and so, as with the behavior of the *Tau* mice under 12L:12D, the local clocks would free-run or exhibit unstable local rhythms. Alternatively, they may adopt a 24 h period, entrained directly by the SCN or indirectly by SCN-dependent feeding rhythms (Izumo et al., 2014; Loh et al., 2015), but the 4 h difference in cell-autonomous periods could only be accommodated by an atypical phase-angle between local and SCN clocks. The greater between-tissue phase-clustering of brain tissues in chimeric vs. WT mice immediately following *ex vivo* culture supports these views.

Across the SCN there are discrete, spatially organized populations of neuropeptidergic cells (and their cognate receptors) distributed within the retinorecipient core, e.g., vasoactive intestinal peptide (VIP) and gastrin-releasing peptide (GRP), or within the shell region, e.g., arginine vasopressin (AVP) and prokineticin 2 (Prok2). Other populations, such as *Drd1a* and neuromedin S (NMS), have a more widespread organization across core and shell (Lee et al., 2015; Smyllie et al., 2016). Using intersectional genetics or targeted gene knockout to manipulate different cell populations has helped us to understand whether it is the nature and identity of targeted cells or the number of cells in a population within SCN that are necessary in driving the circadian period in activity (Lee et al., 2015; Mieda et al., 2015; Smyllie et al., 2016; Hamnett et al., 2020; Patton et al., 2020). In addition to activity, there are reports that the VIP sub-population of cells influence the amplitude of the sleep–wake rhythm, but it is not known whether this is predominantly affecting the level of arousal and/or sleep (Todd et al., 2020). Prokineticin 2 (PK2) may play a role in both circadian and homeostatic regulation of sleep as well as in promoting wakefulness following behavioral challenges (Hu et al., 2007; Jethwa et al., 2008). The delayed transition from sleep to wake in the chimeras observed here under a 10L:10D entraining cycle may result from a loss of coherence between these different neuropeptidergic sub-populations within the SCN, since the *Drd1a*-expressing cells are located in both the core and shell regions of the SCN, resulting in a less coherent/lower amplitude circadian drive to sleep/wake promoting regions, which themselves do have resonance with the environmental 10L:10D cycle (Smyllie et al., 2016).

This delayed switching between sleep–wake following the onset of darkness in the chimera compared to *Tau* mice under 10L:10D could also reflect circadian misalignment between the SCN (24 h) and local circadian clocks (20 h). The sleep–wake regulatory circuit has been described as a flip-flop switch (Saper et al., 2010), and multiple direct and indirect pathways from the SCN to both sleep- and wake-promoting regions of the brain could influence state-switching over the circadian cycle. Even though the chimeric mice were able to entrain to the 10L:10D cycle, and show high amplitude organization to the sleep–wake distribution they showed a significant delay in the transition between sleep to wakefulness. This suggests that the population of 24 h, *Drd1a*-expressing cells in the SCN can promote sleep/inhibit wake, potentially via a direct or indirect influence over neuropeptides such as galanin-positive sleep-promoting cells in the ventro-lateral preoptic area (VLPO), and/or the orexin/GABA-positive wake-promoting cells in the lateral hypothalamus.

Our null hypothesis was that local, non-SCN clocks do not influence sleep and so chimerism would have no effects. Refuting this, chimeras had less NREM sleep over a 24 h LD period/circadian cycle, shorter and more frequent bouts of NREM and a blunted homeostatic response to SD than WT mice. The more fragmented sleep in the chimeras under both LD and DD could not be attributed to the *Tau* genotype because it was not observed in *Tau* controls under DD, as reported in Zhou et al. (2014). Rather, the fragmentation is an emergent feature of circadian chimerism, independent of the singular WT and *Tau* genotypes. Mistimed eating imposed by an enforced misaligned feeding schedule in mice also induced alterations in the 24h distribution of sleep, and greater fragmentation of daytime sleep (Loh et al., 2015). Importantly, however, over 24 h neither total sleep duration, nor fragmentation indices, were altered by misaligned feeding. The phenotype of the chimeric mice in our study, with an internally generated circadian misalignment, is therefore distinct from that associated with externally imposed circadian disturbances.

Another marked difference in the chimeric mice was the blunted recovery from sleep loss. Although the circadian and homeostatic processes appear functionally and neurophysiologically distinct, at a molecular level several components of the circadian timing system have been proposed to play a role in maintaining sleep homeostasis (Franken and Dijk, 2009). For example, global losses of the circadian genes *Cry* and *Bmal1* affect sleep homeostasis (Wisor et al., 2002; Laposky et al., 2005), although in our case the chimeras had competent rather than disabled local clocks. Slow waves associated with NREM sleep and homeostasis are generated within the thalamo-cortical circuits, and are modulated in a circadian manner (Lazar et al., 2015). Chimerism may therefore have affected these circuits by temporally misaligning this local control with SCN-gated behavior. Similarly, the subcortical control of sleep/wake cycles involves a balanced activation between sleep- (e.g., VLPO) and wake-promoting (e.g., LH/TMN) areas in the brain (Weber and Dan, 2016; Scammell et al., 2017); here the 20 h extra-SCN clocks would compromise their interaction with the 24 h SCN as revealed under the 10L:10D photoschedule. In addition, as with forced desynchrony protocols in humans (Wyatt et al., 1999), the importance of the interaction between the homeostatic and circadian processes in regulating sleep/wake is evidenced by the more fragmented sleep in the chimeras, even though in these mice the SCN had the same intrinsic period as the WT. Together, the observed effects on NREM sleep and sleep homeostasis in the chimeras likely reflects a disruption of the local clock mechanisms within and/or between regions important in controlling sleep/wake cycles. Recent evidence has demonstrated that VLPO galanin-expressing neurons are required for consolidated NREMS and contribute to homeostatic regulation of sleep (Ma et al., 2019). This implies that, at a systems-level, there is an important role for the extra-SCN circadian system in influencing both the homeostatic and local processes regulating sleep.

The functional consequences of more fragmented sleep and the blunted homeostatic response in the chimeric mice were examined with a sleep-dependent memory task, NOR. Even though the chimeric mice were entrained, their performance was significantly poorer than that of WT mice. Moreover, it was also worse than that of *Tau* controls, despite the fact that the latter were unable to entrain to the 12L:12D cycle and so the sleep and/or circadian phases were different within and between mice for each phase of the NOR. Although not tested in this study, it remains to be seen whether repeating the NOR in 10L:10D, where both the *Tau* and chimeric mice were entrained, would lead to an improvement in the chimeras’ performance in this task. Successful performance of NOR requires inter-related information on recognition and memorization, which in turn depends on highly integrated circuitries, in particular between the hippocampus and perirhinal cortex (Antunes and Biala, 2012). Performance may have been compromised simply due to fragmented sleep as a consequence of chimerism. Additionally, chimerism may have had a more direct effect on function because of the misalignment between, for example, the hippocampus and the SCN, compromising the timing of local neural and synaptic mechanisms that underpin stimulus discrimination, encoding and recall in the temporal lobe (Ruby et al., 2008; Chuluun et al., 2020).

Our working model is that local clocks direct molecular cycles and organize neural networks in a brain area-specific manner, to facilitate the adaptation of those regions to the contrasting neurobiological requirements of anticipated phases of sleep and of wake. Whereas wake is characterized by online processing of information, sleep is an “off-line” state that, amongst other functions, maintains synaptic homeostasis and consolidation of memory (Cirelli and Tononi, 2020). These incompatible processes of wake and sleep require, and are associated with, very different sets of clock-controlled gene and protein expression. This model is supported in principle, albeit indirectly, by studies of the metabolic impact of circadian misalignment between peripheral organs (Lamia et al., 2008). In a similar way, failure of local clocks to prepare tissues neurochemically for phases of sleep, gated by the SCN, compromises the maintenance of sleep in the chimeric mice. Our results therefore extend our earlier findings from localized ablation of the clock specifically in the TMN, which promotes wakefulness (Yu et al., 2014). This prevented clock-controlled down-regulation of histaminergic tone and consequently impaired the maintenance of NREM sleep. Future studies using targeted chimerism in sleep- or wake-promoting centers, combined with optogenetic circuit manipulation, should provide a more extensive neurochemical dissection of the distributed circadian regulation of sleep.

## Conclusion

Our conclusion that local non-SCN clocks contribute to sleep regulation complements human studies under forced desynchrony (Wyatt et al., 1999) and a study (Muto et al., 2016) where brain activity in response to an attentional task in sleep-deprived individuals was analyzed. It concluded that both the homeostatic and the circadian regulation of the brain are controlled at the local level. The results reported in the present study, where there is a circadian misalignment between the SCN and extra-SCN circadian clocks, underline the importance of cerebral circadian coherence and reveal the consequences of a lack of regional control on both the sleep/wake cycle and cognitive performance. Our results suggest that local tissue clocks and the principal circadian clock in the SCN interact with each other, as well as independently in controlling sleep circuitries. This recognition presents novel opportunities to understand the causes and consequences of sleep and circadian misalignments that are characteristic of numerous diseases, and may suggest new therapeutic strategies.

## Data Availability Statement

The raw data supporting the conclusions of this article will be made available by the authors, without undue reservation.

## Ethics Statement

The animal study was reviewed and approved by MRC Laboratory of Molecular Biology, Animal Welfare and Ethical Review Body (AWERB), United Kingdom.

## Author Contributions

EM and MH: conceptualization. EM and JC: methodology. EM and JC: investigation. EM: formal analysis. NS: preliminary work. EM and MH: writing – original Draft. EM, MH, NS, RW-S, and JC: writing – review and editing. MH: funding acquisition. EM and MH: supervision. All authors contributed to the article and approved the submitted version.

## Conflict of Interest

The authors declare that the research was conducted in the absence of any commercial or financial relationships that could be construed as a potential conflict of interest.
