# Corrigendum: Circadian Chimeric Mice Reveal an Interplay Between the Suprachiasmatic Nucleus and Local Brain Clocks in the Control of Sleep and Memory

**DOI:** 10.3389/fnins.2021.740799

**Published:** 2021-08-05

**Authors:** Elizabeth Susan Maywood, Johanna Elizabeth Chesham, Raphaelle Winsky-Sommerer, Nicola Jane Smyllie, Michael Harvey Hastings

**Affiliations:** ^1^Division of Neurobiology, MRC Laboratory of Molecular Biology, Cambridge, United Kingdom; ^2^Surrey Sleep Research Centre, Faculty of Health and Medical Sciences, University of Surrey, Guildford, United Kingdom

**Keywords:** REM sleep, NREM sleep, electroencephalogram, circadian misalignment, suprachiasmatic nucleus

In the original article, there was an error in assignment of mouse strain identity.

A correction has been made to **Materials and Methods**, **Animals and housing**, **Paragraph number 1**:

All experiments were conducted in accordance with the UK Animals (Scientific Procedures) Act of 1986, with local ethical approval (MRC LMB, AWERB). *Drd1a-Cre* mice (Tg(Drd1-cre)EY266Gsat/Mmucd, RRID:MMRRC_030779-UCD) were purchased from the GENSAT project (Rockefeller University, New York, United States), through the Mutant Mouse Regional Resource Centers (MMRRC, United States). *ROSA-YFP* mice were provided by Dr. A. McKenzie (MRC LMB). Temporally chimeric mice were created by crossing *Drd1a-Cre, ROSA26-EYFP* mice with homozygotes for the floxed *CK1*ε *Tau* allele (Smyllie et al., 2016). All mice expressed the PER2::LUC bioluminescent reporter (Yoo et al., 2005) and had a C57/BL/6J background. This generated four genotypes: CRE-negative, *CK1*ε^*WT*/*WT*^; CRE-positive, *CK1*ε^*WT*/*WT*^; CRE-negative, *CK1*ε^*Tau*/*Tau*^ (*Tau* controls); CRE-positive, *CK1*ε^*Tau*/*Tau*^ (chimera). The first two groups were combined as WT, in light of no differences between them. Males aged 4–6 months old were used to avoid the estrous modulation of activity patterns.

The authors apologize for this error and state that this does not change the scientific conclusions of the article in any way. The original article has been updated.

## Publisher's Note

All claims expressed in this article are solely those of the authors and do not necessarily represent those of their affiliated organizations, or those of the publisher, the editors and the reviewers. Any product that may be evaluated in this article, or claim that may be made by its manufacturer, is not guaranteed or endorsed by the publisher.
